# Forecasting the incidence of mumps in Chongqing based on a SARIMA model

**DOI:** 10.1186/s12889-021-10383-x

**Published:** 2021-02-17

**Authors:** Hongfang Qiu, Han Zhao, Haiyan Xiang, Rong Ou, Jing Yi, Ling Hu, Hua Zhu, Mengliang Ye

**Affiliations:** 1 Department of Epidemiology and Health Statistics, School of Public Health and Management, Chongqing Medical University, Chongqing, 400016 China; 2 Chongqing Municipal Center for Disease Control and Prevention, Chongqing, 400042 China; 3 Department of Medical Informatics Library, Chongqing Medical University, Chongqing, 400016 China

**Keywords:** Incidence, Mumps, SARIMA model, Chongqing

## Abstract

**Background:**

Mumps is classified as a class C infection disease in China, and the Chongqing area has one of the highest incidence rates in the country. We aimed to establish a prediction model for mumps in Chongqing and analyze its seasonality, which is important for risk analysis and allocation of resources in the health sector.

**Methods:**

Data on incidence of mumps from January 2004 to December 2018 were obtained from Chongqing Municipal Bureau of Disease Control and Prevention. The incidence of mumps from 2004 to 2017 was fitted using a seasonal autoregressive comprehensive moving average (SARIMA) model. The root mean square error (RMSE) and mean absolute percentage error (MAPE) were used to compare the goodness of fit of the models. The 2018 incidence data were used for validation.

**Results:**

From 2004 to 2018, a total of 159,181 cases (93,655 males and 65,526 females) of mumps were reported in Chongqing, with significantly more men than women. The age group of 0–19 years old accounted for 92.41% of all reported cases, and students made up the largest proportion (62.83%), followed by scattered children and children in kindergarten. The SARIMA(2, 1, 1) × (0, 1, 1)_12_ was the best fit model, RMSE and MAPE were 0.9950 and 39.8396%, respectively.

**Conclusion:**

Based on the study findings, the incidence of mumps in Chongqing has an obvious seasonal trend, and SARIMA(2, 1, 1) × (0, 1, 1)_12_ model can also predict the incidence of mumps well. The SARIMA model of time series analysis is a feasible and simple method for predicting mumps in Chongqing.

## Background

Mumps is a disease caused by an infection due to mumps virus. It is a vaccine-preventable toxic disease in children [1], and the main population affected is children and adolescents [2]. The clinical manifestation of mumps virus infection is pain and swelling of the parotid gland, but it may also affect various tissues and organs [3]. It can also cause serious complications, such as encephalitis, meningitis, orchitis, myocarditis, pancreatitis, and nephritis [3, 4]. Patients generally recover spontaneously within a few moments of infection, but the disease has long-term consequences, such as seizures, cerebral palsy, hydrocephalus and deafness [4, 5]. Mumps is a global epidemic [6], with outbreaks occurring in several regions, such as Ireland [7], Nebraska [8], and Arkansas [9]. The incidence of mumps in China is high [10].

In 2018, China had the highest number of cases in the world (259,071 cases), followed by Nepal (29,614 cases) and Burkina Faso (26,982 cases) [11]. From 2004 to 2018, China reported 4,272,368 cases, and the average incidence was 2144 per 100,000 per year [12]. In 1990, mumps was included in the management of class C infectious diseases (class C infectious diseases are known as surveillance and management infectious diseases, including filariasis, hydatid disease, leprosy, influenza, mumps, epidemic and endemic typhus, rubella, acute hemorrhagic conjunctivitis, hand, foot and mouth disease, and infectious diarrhoeal diseases other than amoebic dysentery, typhoid and paratyphoid, etc.) [13]. Mumps-containing vaccines were included in the expanded national immunization program in 2008 [14]. From 2014 to 2016, the reported incidence of mumps began to decline nationwide, but rose again in 2017 and 2018 [12]. Mumps is highly contagious and often causes outbreaks in school nurseries and other collective units, seriously affecting the normal school teaching order. Mumps is one of the important public health problems that endanger the physical and mental health of children and adolescents in China [15]. Thus, understanding the epidemic regularity and predicting the epidemic trend of mumps is crucial for risk analysis and health resource allocation in the health sector.

Time series analysis is a scientific quantitative prediction of the future trend of diseases based on historical data and time variables. It is a quantitative analysis method that does not consider the influence of complex factors [16]. The ARIMA model is one of the most representative and widely used models in time series prediction [17], and this method is simple and requires only endogenous variables instead of other exogenous variables. In epidemiological studies, the ARIMA model has been used in many studies, such as malaria [18], tuberculosis [19, 20], dengue [21] and other diseases [22, 23]. To the best of our knowledge, Chongqing is one of the areas with the highest incidence rates in China [24]. Furthermore, no previous research has been conducted to predict the incidence of mumps in Chongqing. To address these noted gaps, this paper is the first to establish a time series analysis of the mumps incidence data for short-term prediction in Chongqing. We developed a seasonal autoregressive comprehensive moving average (SARIMA) model to forecast the incidence of mumps. As we all know, the SARIMA model has one more seasonal effect than the ARIMA model, and it is generally handled by seasonal difference [25]. The results of this study may be useful for predicting mumps epidemics and offer reference information for mumps control and intervention in Chongqing.

## Methods

### Data collection

In this study, mumps data from 2004 to 2018 were collected from Chongqing CDC. All cases in each region were verified through clinical and laboratory diagnosis, and reported to the CDC by the health department. The reported data includes the sex, occupation, age and region of the patient. The data were finally collected from each region and submitted to the Chongqing CDC.

### SARIMA model construction

SARIMA differs from ARIMA models in that it contains seasonal characteristics of time series [26], and is an extension of ARIMA model. The general structure of SARIMA model is expressed as SARIMA (p, d, q) × (P, D, Q)_*S*_, and its formula is as follows [27]:

$$ {\displaystyle \begin{array}{l}{\nabla}^d{\nabla}_S^D{x}_t=\frac{\Theta (B){\Theta}_S(B)}{\Phi (B){\Phi}_S(B)}{\varepsilon}_t\\ {}\Theta (B)=1-{\theta}_1B- ggg-{\theta}_q{B}^q\\ {}\Phi (B)=1-{\phi}_1B- ggg-{\phi}_p{B}^p\\ {}{\Theta}_S(B)=1-{\theta}_1{B}^S- ggg-{\theta}_Q{B}^{QS}\\ {}{\Phi}_S(B)=1-{\phi}_1{B}^S- ggg-{\phi}_P{B}^{PS}\end{array}} $$

In the above equation, B represents the backward shift operator, ε_*t*_ denotes the residual at time t, the mean of ε_*t*_ is zero and the variance of ε_*t*_ is constant, *x*_*t*_ is the observed value at time t (t = 1, 2 … k). In SARIMA (*p*, *d*, *q*) × (*P*, *D*, *Q*)_*S*_, s is the length of the seasonal period, p, P, d, D, q and Q are the autoregressive order, seasonal autoregressive order, number of difference, number of seasonal difference, moving average order and seasonal moving average order, respectively [27]. According to the sequence autocorrelation function (ACF) and partial autocorrelation function (PACF) for determining the values of the six parameters in the SARIMA model. Akaike information criterion (AIC) and Schwarz Bayesian Criterion (BIC) are two indexes of model optimization. A small AIC shows that is the better fitting model [26].

Finally, two indexes were used to compare the fitting effect. The formulas for RMSE and MAPE are [28]:

$$ RMSE=\sqrt{\frac{1}{n}\sum \limits_{t=1}^n{\left({x}_t-\overset{\wedge }{x_t}\right)}^2} $$

$$ MAPE=\frac{1}{n}\sum \limits_{t=1}^n\frac{\mid {x}_t-\overset{\wedge }{x_t}\mid }{x_t} $$

In the above equation, x_t_ is the actual incidence value, $\overset{\wedge }{x_t}$ is the estimated incidence value, n is the number of months for forecasting. The lower RMSE and MAPE value, the better the data fitting effect.

### Statistical analysis

Firstly, we used Excel 2010 to conduct a descriptive analysis of mumps in Chongqing from 2004 to 2018, and explain the sex, age, and occupational distribution of the disease onset. Secondly, the time series analysis of the mumps incidence sequence was analyzed. The “stl” function in the R 3.5.0 software was used to decompose the seasonal trend of the sequence. Finally, under the operation of the R3.5.0 software, a SARIMA model was established to predict the incidence of mumps. In this study, the incidence of mumps from 2004 to 2017 was used as a training dataset to fit the SARIMA model, predict the incidence of mumps in 2018, and verify the predicted effect. The significance level was *p* < 0.05.

## Results

**Fig. 1:**
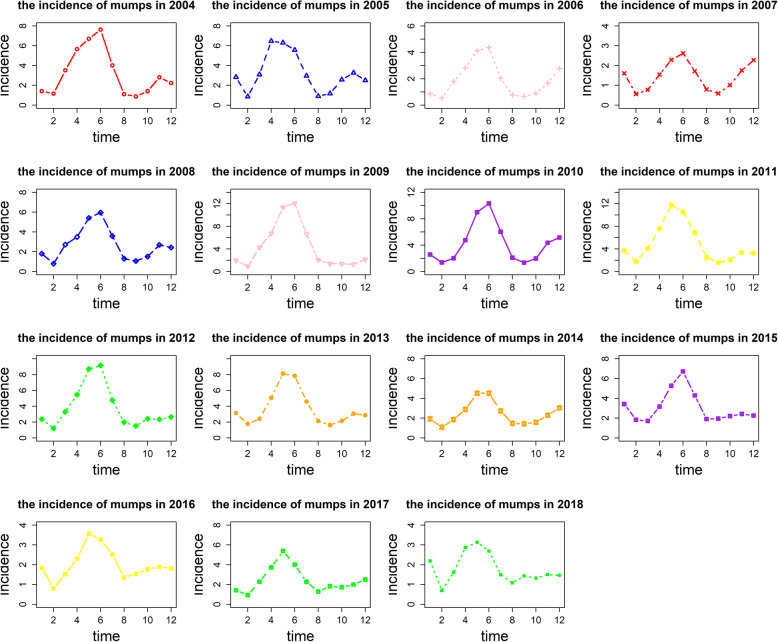
Monthly incidence of mumps from 2004 to 2018 in Chongqing

The monthly incidence of mumps number is presented in Fig. 1, showing that monthly mumps incidence was low in February and peaked in April to July. Table 1 shows the top five regions with the highest number of mumps in Chongqing in the past 15 years. Although the location of Chongqing has changed, it is mainly concentrated in the northeast and west of Chongqing. Table 2 shows reported 159,181 mumps cases in the past 15 years (2004–2018), in Chongqing, The cases included 93,655 males and 65,526 females (male-to-female ratio of 1.43:1), with mumps commonly occurring between the ages of 0 and 19, and the age group of 0–19 years accounted for the 92.41% (*n* = 147,094) of all reported cases. The group with the highest proportion of mumps is students, amount to 62.83% (*n* = 100,013), followed by scattered children and children in kindergarten.

**Table 1:** The top five regions with more mumps cases each year in Chongqing from 2004 to 2018

Time	No 1	No 2	No 3	No 4	No 5
2004	Changshou District	Jiulongpo District	Hechuan	Nanan District	Yubei District
2005	Jiangjin District	Hechuan	Kaixian	Fuling District	Wanzhou District
2006	Nanchuan	Banan District	Shapingba District	Fengdu	Beibei District
2007	Shapingba District	Beibei District	Dianjiang	Yunyang	Tongnan
2008	Wanshneg District	Dianjiang	Shapingba District	Jiangjin District	Kaixian
2009	Kaixian	Wanzhou District	Jiangjin District	Hechuan	Jiulongpo District
2010	Wanzhou District	Jiulongpo District	Yongchuan District	Yunyang	Yubei District
2011	Changshou District	Dainjiang	Beibei District	Jiulongpo District	Yubei District
2012	Wushan	Yubei District	Jiulongpo District	Miao-Tujia Autonomous County of Pengshui	Shapingba District
2013	Kaixian	Wanzhou District	Jiangjin District	liangping	Shapingba District
2014	Wanzhou District	Zhongxian	Nanan District	Shapingba District	Kaixian
2015	Yubei District	Nanan District	Changshou District	Yongchuan District	Hechuan
2016	Yubei District	Dazu District	Wulong	Yongchuan District	Jiangjin District
2017	Jiangjin District	Jiulongpo District	Yubei District	Nanan District	Tongliang
2018	Jiulongpo District	Yubei District	Nanan District	Yongchuan District	Shapingba District

**Table 2:** Distribution of mumps by sex, age and occupation in Chongqing from 2004 to 2018

**Variable**	**Total**	**2004**	**2005**	**2006**	**2007**	**2008**	**2009**	**2010**
	***N*** **= 159,181(%)**	***n*** **= 12,059(%)**	***n*** **= 10,741(%)**	***n*** **= 6519(%)**	***n*** **= 4927(%)**	***n*** **= 9224(%)**	***n*** **= 14,814(%)**	***n*** **= 14,575(%)**
Sex								
Male	93,655(58.84)	7175(59.5)	6474(60.27)	3879(59.50)	2922(59.31)	5593(60.64)	8908(60.13)	8612(59.00)
Female	65,526(41.16)	4884(40.5)	4267(39.73)	2640(40.50)	2005(40.69)	3631(39.36)	5906(39.87)	5963(40.91)
Age. years								
0–9	101,693(63.89)	7899(65.5)	6780(63.12)	4078(62.56)	3116(63.24)	5536(60.02)	8694(58.69)	9430(64.70)
10–19	45,401(28.52)	3386(28.08)	3427(31.91)	2105(32.29)	1452(29.47)	3067(33.25)	5120(34.56)	4049(27.78)
20–29	4684(2.94)	340(2.82)	210(1.96)	137(2.10)	155(3.15)	279(3.02)	412(2.78)	435(2.98)
30–39	3913(2.46)	317(2.63)	206(1.92)	129(1.98)	116(2.35)	221(2.40)	389(2.63)	416(2.85)
40–49	1719(1.08)	56(0.46)	55(0.51)	29(0.44)	42(0.85)	47(0.51)	103(0.70)	129(0.89)
≥ 50	1771(1.11)	61(0.51)	63(0.59)	41(0.63)	46(0.93)	74(0.80)	96(0.65)	116(0.80)
Occupation								
Workers	1491(0.94)	154(1.28)	74(0.69)	35(0.54)	52(1.06)	89(0.96)	131(0.88)	146(1.00)
Housework and unemployment	2264(1.42)	106(0.88)	69(0.64)	52(0.80)	43(0.87)	101(1.09)	148(1.00)	201(1.38)
Scattered children	12,785(8.03)	821(6.81)	622(5.79)	548(8.41)	434(8.81)	932(10.10)	1406(9.49)	1302(8.93)
Children in kindergarten	33,901(21.30)	1506(12.49)	1088(10.13)	981(15.05)	1036(21.03)	1844(19.99)	3117(21.04)	3650(25.04)
Students	100,013(62.83)	8930(74.05)	8516(79.28)	4638(71.15)	3107(63.06)	5827(63.17)	9280(62.64)	8491(58.26)
Others	8727(5.48)	542(4.49)	372(3.46)	265(4.07)	255(5.18)	431(4.67)	732(4.94)	785(5.39)
**Variable**	**2011**	**2012**	**2013**	**2014**	**2015**	**2016**	**2017**	**2018**
	***n*** **= 17,061(%)**	***n*** **= 13,388(%)**	***n*** **= 13,188(%)**	***n*** **= 8710(%)**	***n*** **= 11,113(%)**	***n*** **= 7301(%)**	***n*** **= 8946(%)**	***n*** **= 6615(%)**
Sex								
Male	10,102(59.21)	8011(59.84)	7556(57.29)	4984(57.22)	6326(56.92)	4310(59.03)	5070(56.67)	3733(56.43)
Female	6959(40.79)	5377(40.16)	5632(42.71)	3726(42.78)	4787(43.08)	2991(40.97)	3876(43.33)	2882(43.57)
Age. years								
0–9	10,955(64.21)	9101(67.98)	9094(68.96)	5642(64.78)	7183(64.64)	4917(67.35)	5338(59.67)	3930(59.41)
10–19	4626(27.11)	3212(23.99)	2965(22.48)	2280(26.18)	2977(26.79)	1754(24.02)	2892(32.33)	2089(31.58)
20–29	609(3.57)	399(2.98)	421(3.19)	283(3.25)	349(3.14)	208(2.85)	240(2.68)	207(3.13)
30–39	511(3.00)	372(2.78)	326(2.47)	201(2.31)	242(2.18)	149(2.04)	188(2.10)	130(1.97)
40–49	198(1.16)	180(1.34)	210(1.99)	157(1.80)	180(1.62)	133(1.82)	114(1.27)	86(1.30)
≥ 50	162(0.95)	124(0.93)	172(1.30)	147(1.69)	182(1.64)	140(1.92)	174(1.95)	173(2.62)
Occupation								
Workers	203(1.19)	124(0.93)	108(0.82)	91(1.04)	98(0.88)	58(0.79)	83(0.93)	45(0.68)
Housework and unemployment	259(1.52)	200(1.49)	242(1.84)	158(1.81)	272(2.45)	107(1.47)	167(1.87)	139(2.10)
Scattered children	1751(10.26)	1315(9.82)	1044(7.92)	595(6.83)	613(5.52)	522(7.15)	479(5.35)	401(6.06)
Children in kindergarten	4092(23.98)	3543(26.46)	3887(29.47)	1973(22.65)	2226(20.03)	1648(22.57)	1675(18.72)	1635(24.75)
Students	9634(56.27)	7343(54.85)	7092(53.78)	5327(61.16)	7273(65.45)	4495(61.57)	6070(67.85)	3990(60.32)
Others	1122(6.58)	863(6.45)	815(6.18)	566(6.50)	631(5.68)	471(6.45)	472(5.28)	405(6.12)

### SARIMA (p, d, q) × (P, D, Q)_s_ model

**Fig. 2:**
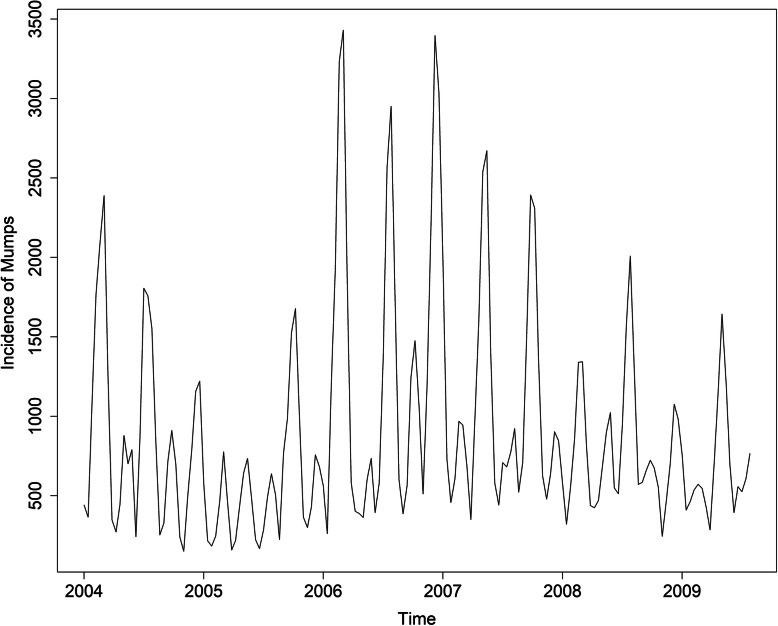
Reported monthly incidence of mumps from January 2004 to December 2017

**Fig. 3:**
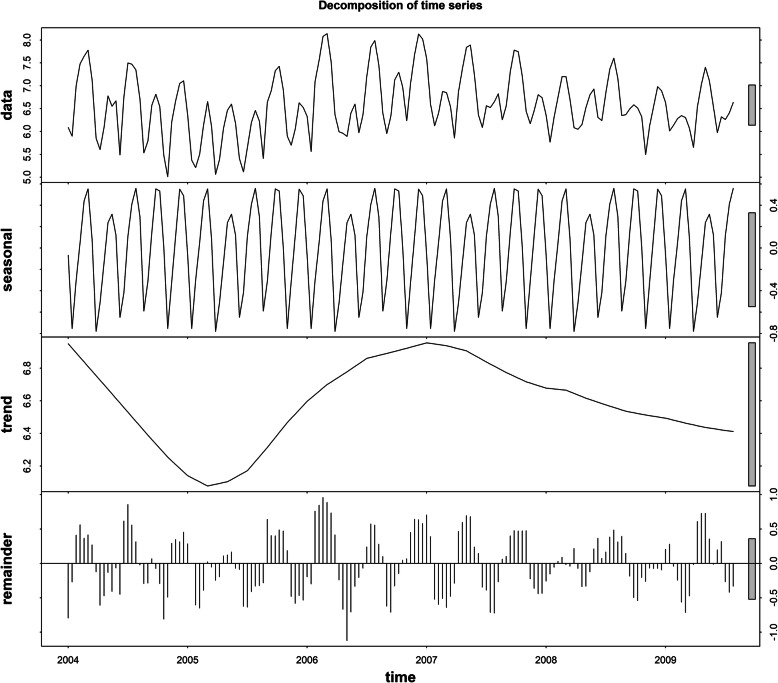
Trend, seasonal and residual components derived from STL decomposition of monthly mumps incidence for Chongqing during 2004–2018

**Fig. 4:**
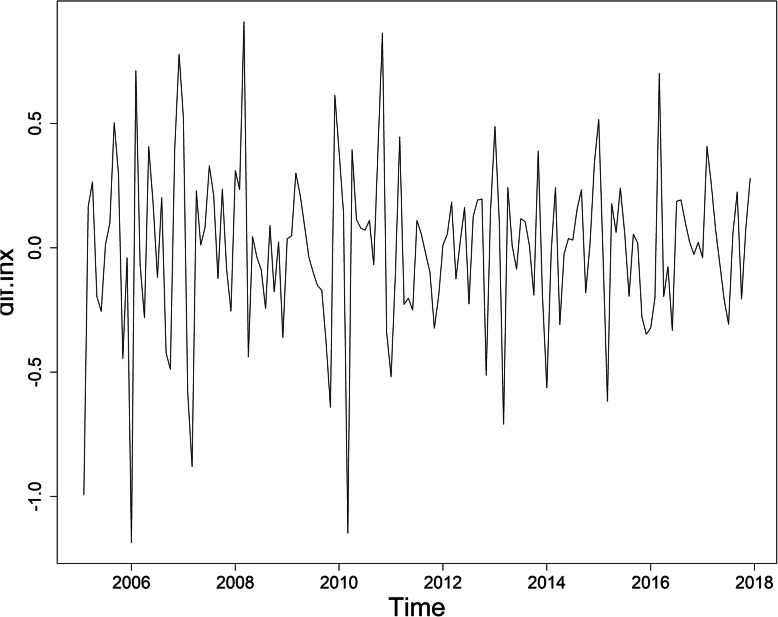
Sequence diagram after a 1-step difference and seasonal difference with a period of 12

**Fig. 5:**
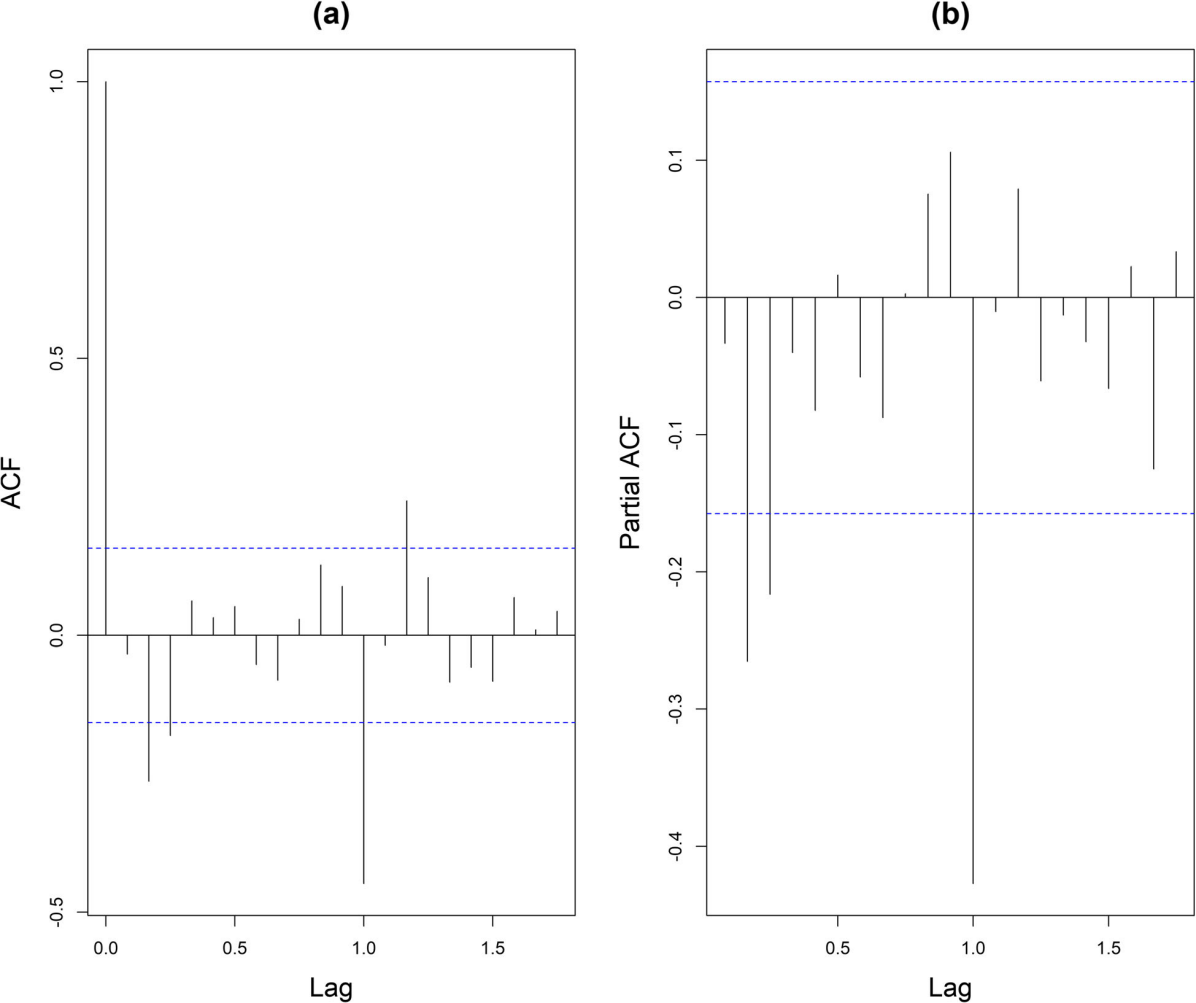
Autocorrelation function (ACF) and partial ACF charts of monthly mumps incidence numbers. **a** ACF chart; **b** Partial ACF chart

Figure 2 shows the sequence diagram of mumps, and the sequence is nonstationary. Furthermore, the monthly incidence of mumps in Chongqing suggested a slightly decreasing tendency and seasonal tendency. Figure 3 shows the decomposition diagram of the sequence, with an obvious seasonality. After the natural logarithm transformation of the original data, a one-step difference and seasonal difference with a period of 12 was conducted to remove non-stationarity. The sequence diagram after the difference was stationary (Fig. 4), and the ADF test results showed that the sequence was stationary (*p* < 0.05). Figure 5 shows that ACF and PACF of the sequence were both trailing. Considering that the value of p, q, P and Q generally does not exceed 2, a trial order from 0 to 2 was performed. Only five models passed the test and the model parameter test: SARIMA(1, 1, 1) × (1, 1, 0)_12_, SARIMA(0, 1, 2) × (1, 1, 0)_12_, SARIMA(1, 1, 1) × (0, 1, 1)_12_, SARIMA(2, 1, 1) × (0, 1, 1)_12_, SARIMA(1, 1, 2) × (0, 1, 1)_12_. The AIC, BIC values, and two error indicators of the five models were compared in Table 3. And the SARIMA(2, 1, 1) × (0, 1, 1)_12_ model was selected as the best one.

**Table 3:** AIC values, BIC values, RMSE and MAPE for different SARIMA models

	AIC	BIC	Goodness of Fits for Models	
			RMSE	MAPE(%)
SARIMA(1,1,1)(1,1,0)_12_	57.34	69.52	0.2686	41.2841
SARIMA(0,1,2)(1,1,0)_12_	61.93	74.11	0.2734	44.7352
SARIMA(1,1,1)(0,1,1)_12_	34.42	46.60	0.2450	40.5910
SARIMA(2,1,1)(0,1,1)_12_	30.05	45.27	0.2392	44.3186
SARIMA(1,1,2)(0,1,1)_12_	31.14	46.36	0.2402	43.0071

Table 4 shows the estimated and standard errors of model parameters and their corresponding significance values. The model equation is given as

$$ \nabla {\nabla}^{12}{x}_t=\frac{1-0.8197B}{1-0.7186B+0.2305{B}^2}\left(1-0.7643{B}^{12}\right){\varepsilon}_t\kern0.5em ,{\varepsilon}_t\sim N\left(0,0.062\right) $$

**Table 4:** Estimates and standard error of SARIMA(2, 1, 1) × (0, 1, 1)_12_ model parameters

Measurements	Model Terms	Estimates	Standard Error	t-Value	*p*-Value
Non-Seasonality	AR1 term	0.7186	0.1107	6.29	*P* < 0.05
	AR2 term	−0.2305	0.0878	2.63	*P* < 0.05
	MA1 term	−0.8197	0.8960	9.15	*P* < 0.05
Seasonality	Seasonality MA1	−0.7643	0.0694	11.01	*P* < 0.05

**Fig. 6:**
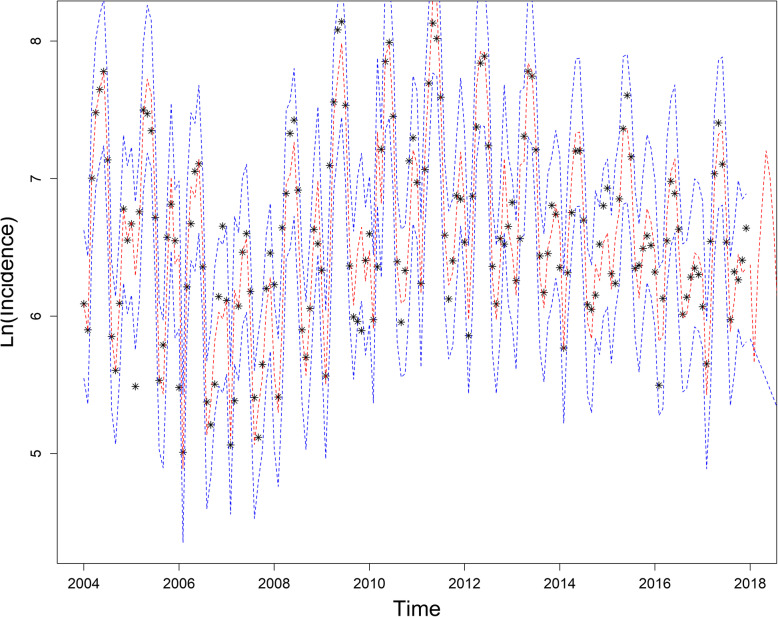
SARIMA(2, 1, 1) × (0, 1, 1)_12_ model fitting, verification and forecasting of mumps incidence in Chongqing from January 2004 to December 2018. The black asterisk represents the actual value, the red dotted line represents the fitting value, and the blue dotted line represents the 95% confidence interval.  Actual Data; SARIMA Model Fitting Values;  95%Confidence Line

The SARIMA(2, 1, 1) × (0, 1, 1)_12_ model was used to forecast the incidence of mumps in 2018. Table 5 shows the value of prediction, RMSE and MAPE values are 0.9950 and 39.8396%, respectively. The actual value of incidence and fitted incidence of SARIMA model monthly are shown in Fig. 6. Figure 6 and Table 5 show that the tendency and epidemics from predicted incidence are close to actual value of incidence and epidemic circumstance of mumps.

**Table 5:** Reported and forecasted incidence of mumps for 2018

Time (Month)	Actual incidence	Forecasted incidence
January 2018	2.1885	2.0852
February 2018	0.6959	1.0585
March 2018	1.6259	1.9447
April 2018	2.8616	3.2639
May 2018	3.1381	5.0355
June 2018	2.6958	4.8205
July 2018	1.4959	2.9731
August 2018	1.0796	1.4132
September 2018	1.4373	1.4198
October 2018	1.3203	1.6622
November 2018	1.5056	2.0661
December 2018	1.4666	2.2634

## Discussion

In this study, we found that the annual incidence of mumps decreased significantly from 2004 to 2007 and increased from 2007 to 2011. The lowest incidence was in 2007 (175,463/100000) and the highest was in 2011 (591,471/100000). In the 159,181 reported cases, males were 1.43 times as many as females. These findings are consistent with the results of relevant literature [29]. In terms of age and occupation, the largest proportion of mumps cases were 0–19 years old (92.41%) and students (62.83%), indicating that children and students are the main targets of protection. In addition, this study shows that the western and northeastern of Chongqing are high-incidence areas (Table 1). Therefore, the government should strengthen the prevention and control measures of mumps in important areas and populations.

We decomposed the mumps incidence sequence and found that the mumps incidence sequence had obvious trends and seasonality, the monthly incidence of mumps was low in February and peak in April to July, which was consistent with previous studies [10, 30]. Therefore, taking some interventions are necessary to reduce the spread of infectious diseases in public transportation from April to July.

The results of this study indicate that model *SARIMA*(2, 1, 1) × (0, 1, 1)_12_ is the best predictive model. Figure 6 shows the 95% CIs of the forecast value in this paper containing all of the real observed data, which is a good match between the observed value and the fitted value. The incidence of mumps in 2018 peaked from April to June but showed a decreasing trend after June due to the reduction of students’ contact during the summer vacation. Thus far, in terms of infectious diseases, the SARIMA model has good results in predicting hand, foot, and mouth disease [31] and tuberculosis [32]. This study is the first to use the SARIMA model to analyze the incidence of mumps in Chongqing. The *SARIMA*(2, 1, 1) × (0, 1, 1)_12_ model in this study can well reflect the incidence of mumps in Chongqing, and has a good short-term predictive effect. It can provide early warning to health authorities to formulate plans and implement public health intervention measures to prevent and control the disease.

Compared with the ARIMA model, the SARIMA model increases the seasonal effect and is suitable for analyzing sequences with obvious seasons and periodicity [33], while most epidemiological data are seasonal and periodic [32, 34–36]. Compared with other time series analysis methods, The SARIMA model generally adopts the logarithm method, difference method and the seasonal difference method [37, 38] to make the series stable without the need for complicated conversion or variable substitution [39, 40]. In addition, related research shows that among various time series analysis methods, ARIMA is a useful tool for interpreting surveillance data for disease prevention and control [41, 42].

This study has several limitations. We only made short-term predictions. Therefore, various factors affecting the incidence of mumps should be considered to establish a long-term and stable prediction model. In future research, hybrid models, such as the SARIMA-NAR hybrid model [43], SARIMA-NARNNX hybrid model [44], and SARIMA-NARX hybrid model [45], can be used to analyze or predict diseases.

## Conclusions

The results of this study suggest that applying the ARIMA time series models to forecast the incidence of mumps is feasible. The confidence intervals of the predicted values contain all the actual values. Thus, the SARIMA(2, 1, 1) × (0, 1, 1)_12_ model may be used to predict the incidence of mumps. The short-term prediction of mumps is effective, which is helpful for the evaluation of prevention or control measures. Meanwhile, timely and effective countermeasures can be adopted for the epidemic peak that may occur. For instance, the government has strengthened publicity on mumps prevention and control knowledge to improve the public’s awareness of the disease.

## Data Availability

The data that support the findings of this study are available from the Chongqing Municipal Center for Disease Control and Prevention, but restrictions apply to the availability of these data, which were used under license for the current study, and so are not publicly available. Data are however available from the authors upon reasonable request and with permission of Chongqing CDC.

## Abbreviations

SARIMA: Seasonal autoregressive integrated moving average
RMSE: Root mean square error
MAPE: The mean absolute percentage error
AIC: Akaike Information Criterion
BIC: Schwarz Bayesian Criterion
